# Relation between personality dimensions and symptomatology of depression in skin cancer patients

**DOI:** 10.1186/s13030-021-00220-3

**Published:** 2021-10-12

**Authors:** ML Ramírez-de los Santos, A López-Navarro, S Ramírez-de los Santos, JM Guzmán-Flores, AL Pereira-Suárez, EI López-Pulido

**Affiliations:** 1 Centro Universitario de los Altos, Universidad de Guadalajara, Road to Yahualica 7.5 Km, Jalisco 47600 Tepatitlan de Morelos, Mexico; 2 Centro Universitario de Ciencias de la Salud, Universidad de Guadalajara, Guadalajara, Jalisco Mexico

**Keywords:** Personality dimensions, Anxiety, Depression, Skin cancer

## Abstract

**Background:**

Environmental psychological factors such as mood states can modify and trigger an organic response; depressive disorder is considered a risk factor for oncological development, leading to alterations both in the genesis and in the progression of the disease. Some authors have identified that personality relates to mood since a high score in neuroticism is associated with intense and long-lasting emotions of stress and therefore with the development of depressive behaviors. The objective of this study was to analyze the relationship between personality and depression in skin cancer patients.

**Methods:**

A total of forty-seven clinically and histopathologically diagnosed patients were scheduled for an hour-long interview, during which they provided informed consent and sociodemographic information. The psychological questionnaires applied were the revised Eysenck Personality Questionnaire and the clinical questionnaire for the diagnosis of the depressive syndrome.

**Results:**

The patient’s mean age was 66.5 years (SD ± 12.4) and the majority were diagnosed with basal cell carcinoma (70.2%). The frequency of anxious/depressive symptoms was 42.5%, with an increase in depression scores in the female gender (*p* < 0.001). Furthermore, a difference was found in the neuroticism dimension related to gender, with higher values in women (*p* = 0.002). Depressive symptomatologic portraits were correlated with the dimensions of neuroticism (*p* < 0.001, r = 0.705), psychoticism (*p* = 0.003, r = 0.422) and lying (*p* = 0.028, r = − 0.321).

**Conclusions:**

Our results support the hypothesis that personality dimensions are related to the presence of anxiety/depressive symptomatology in patients with skin cancer, especially in the female gender. Highlighting the need for future research that delves into the implications at the psychological level, the quality of life, and the biological mechanisms that link personality and depressive symptoms in the development and evolution of skin cancer.

## Background

The personality plays an important role not only in the reaction to stressful events, but it also influences the presence of various types of predominant emotions in the person. Such is the case of neuroticism where high levels of it have been identified as a risk factor for the development of depressive disorder [1, 2]. Depression occurs in various forms and contains a wide range of symptoms characterized mainly by anhedonia and low moods [3, 4]. It is a public health problem, reported as the third cause of disability worldwide and its presence is associated with various chronic inflammatory diseases [5–8].

Depression in cancer patients is very common and its incidence rate is up to three times higher than in the general population, it has been reported to affect more than 10% of patients and to a greater extent in women [9–12]. However, despite being frequent, on repeated occasions, depressive symptoms in clinical practice are not identified. Concerning skin cancer, a high prevalence of depressive symptoms has been observed in patients with basal cell carcinoma (BCC) because the quality of life of individuals after the diagnosis of the disease declines considerably [13–15]. Also, a higher prevalence of anxiety and depression has been identified in patients with advanced melanoma compared to those with early disease [16].

One psychosocial construct inviting debate is that of personality and its possible role in cancer outcomes. As personality traits are believed to be stable, they would seem more as transient states in the prediction of disease progression over longer periods [17, 18]. In patients with squamous cell carcinoma (SCC), a relationship has been observed with scores of the neuroticism dimension [19, 20]. Regarding melanoma skin cancer, a possible association with the extraversion and psychoticism personality dimensions has been identified [21].

The objective of this study is to describe personality dimensions and the prevalence of anxiety or depression symptomatology in a skin cancer population. Following, we analyze the data looking for a possible relation between the personality and depression scores.

## Methods

### Study population

The sample consisted of forty-seven Mexican skin cancer patients who attend a private dermatological consultation. All patients were included at an early stage (stage I) according to the American Joint Commission on Cancer (AJCC) TNM system. Since BCC, SCC, and mixed carcinoma no presented high-risk features, tumors were less than 2 cm and have not spread to nearby lymph nodes or organs. Melanomas were localized, smaller than 1 mm in depth and not ulcerated. The sampling method used was non-probabilistic for convenience, limiting the selection of subjects by geographical proximity and that they met the inclusion criteria, achieving a homogeneous population to minimize the required number of participants. Requirements to be part of the project included age of 18 years or older, clinical, and histopathological diagnosis of skin cancer, and sign the informed consent. Within the exclusion criteria, pregnancy, the presence of some fungal disease or other types of concomitant acute infection, and other inflammatory diseases were considered. The study was developed according to the guidelines of the Helsinki Declaration World Medical Association (Helsinki Declaration World Medical Association, 2013) with the approval of the Clinic department and institutional bioethics committee at the Guadalajara University (CUA/CEI/DOBI008/2021).

### Data collection procedure

Clinically and histopathologically diagnosed patients were scheduled for an hour-long interview, during which they provided informed consent and sociodemographic information. The psychological questionnaires were also applied: EPQ-R (full version) and the clinical questionnaire for the diagnosis of the depressive syndrome.

### Measures

*Sociodemographic information.* Information related to age, gender, marital status, employment status was collected along with medical information as the diagnosis, body site, and history of non-melanoma skin cancer (NMSC).

#### Personality dimensions

The EPQ-R questionnaire was used to measure the personality dimensions: extraversion (E), neuroticism (N), psychoticism (P), and social desirability or lying (L). It consists of 83 items that correspond to four dimensions with dichotomous responses. The questionnaire is interpreted based on each of the dimensions, which yield a score as they increase or decrease, indicating greater or lesser presence or strength of that personality. The Cronbach coefficient considered to be a measure of scale reliability was calculated for our sample with a value of α = 0.75, indicating acceptable internal consistency [22].

#### Depression

The clinical questionnaire was used to evaluate the symptoms and diagnose the mood: normal, anxiety reaction, depression of medium intensity, and severe depression [23]. It consists of 20 items, corresponding to common symptoms present in depression, with responses on a Likert-type scale with values ​​from 1 to 4 according to intensity, obtaining minimum scores of 20 and a maximum of 80. The results are interpreted based on the scores, from 20 to 35 points are considered normal moods, from 36 to 45 are identified as patients with anxiety reactions, from 46 to 65 are considered as patients with depressive symptoms of medium intensity, and finally, if the patient presents scores of 66–80 it is considered that he/she presents a severe depressive state. Regarding the reliability of the questionnaire, Cronbach’s alpha was calculated for our sample and a value of α = 0.86 was obtained, suggesting a good internal consistency [22].

### Data analysis

Descriptive statistics were calculated for sociodemographic data, personality dimensions scores, and depression frequency. The influence of gender and age on personality and depression was determined using the Student T-test or Mann-Whitney U test. Other sociodemographic and clinical variables such as marital status, occupation, phototype, and skin cancer diagnosis were evaluated by the Kruskal-Wallis test and one-way ANOVA. Finally, Correlations were determined by Pearson or Spearman coefficients to examine the relationship between the EPQ-R and depression questionnaire measures. The analyses were conducted using SPSS 22 software with a significance level set at *p* < 0.05.

## Results

### Sociodemographic and clinical characteristics

According to the sociodemographic data, the mean age of the patients included was 66.5 years (SD ± 12.4). Patients of both genders were incorporated in a similar proportion. Regarding marital status, more than half of them were married people (72.4%), in the interview, the participants referred to various occupations, one of the most cited was that of a housewife (48.9%). About the Fitzpatrick skin phototype scale (FST), most skin cancer patients presented II and III phototypes (93.7%). On the other hand, with the support of both private practice and the Dermatological Institute of Jalisco “Dr. José Barba Rubio”, certified by the Mexican Council of Dermatology and The International Committee for Dermatopathology, it was identified that 70.2% of the patients presented BCC, followed by SCC (23.4%). Meanwhile, melanoma (4.3%) and mixed carcinoma (2.1%). All these malignant neoplasms were mainly located in the head and neck area (68.1%) and the recurrence reported to date occurs in almost a fifth of the population captured (19.1%). Table 1 shows the complete values ​​of each of the sociodemographic variables mentioned above.

**Table 1:** Demographic and clinical characteristics of the study population

Characteristic		*n*	Percentage
*Age*	30–59	10	21.3
	60–90	37	78.7
	*Total*	47	100
*Gender*	Female	26	55.3
	Male	21	44.7
	*Total*	47	100
*Marital status*	Single	7	14.9
	Married	34	72.4
	Divorced	1	2.1
	Widowed	5	10.6
	*Total*	47	100
*Occupation*	Farm workers	13	27.7
	Home maker	23	48.9
	Retired	7	14.9
	Other	4	8.5
	*Total*	47	100
*Fitzpatrick Phototypes*	I	2	4.2
	II	24	51.2
	III	20	42.5
	IV	1	2.1
	*Total*	47	100
*Diagnosis of skin cancer*	Melanoma	2	4.3
	BCC	33	70.2
	SCC	11	23.4
	BCC/SCC	1	2.1
	*Total*	47	100
*Location*	Head/Neck	32	68.2
	Back/chest	0	0
	Limbs (arms/legs)	2	4.2
	Missing	13	27.6
	*Total*	47	100
*Recurrence*	Yes	9	19.1
	No	28	59.6
	Missing	10	21.3
	*Total*	47	100

Notes: *BCC* basal cell carcinomas, *SCC* squamous cell carcinomas

### Personality profile and symptomatology of depression

Regarding the psychological questionnaire (EPQ-R) which includes the personality dimensions neuroticism, extraversion, psychoticism, and lying or dissimulation was applied to the patients. In the first place, the number of items corresponding to the dimensions was identified, as well as the minimum scores and maximums obtained. After, the mean and standard deviation were calculated which corresponded to those reported in studies from the same test in the Spanish population. However, an increase of the L dimension means scores to the mean used by the instrument was identified. The complete values are observed in Table 2.

**Table 2:** Skin cancer patients scores on the EPQ-R

Variable	Items	Mean	SD	Minimum	Maximum
Extraversion	19	11.6	4.7	3	19
Neuroticism	23	11.8	6.6	0	23
Psychoticism	23	6.9	2.6	0	14
Lie	18	14.3	2.8	5	18

Note: *SD* Standard deviation

Relating to the analysis of the distribution by frequencies of the questionnaire for the diagnosis of depressive symptoms, they were classified as normal patients when they did not show symptoms, anxiety reaction, medium intensity depression, or severe state of depression. Where we find the presence of depressive symptomatology in almost half of the patients (42.5%), corresponding to anxiety reaction and medium intensity depression, as shown in Table 3.

**Table 3:** Diagnosis of depressive symptomatology in skin cancer patients

Diagnostic	Frequency	Percentage
*Normal*	27	57.4
*Anxiety reaction*	11	23.4
*Medium intensity depression*	9	19.1
*Severe state of depression*	0	0
*Total*	47	100

Later we evaluate the influence of gender and age on the personality and depression scores. Where it was observed that the female gender presents higher values in the dimension of N (*p* = 0.002), while the dimensions of E, P, and L were perceived as similar both in men as in women. When making comparisons by age groups, elevated values of L dimension are observed in the 60–90 age group (*p* = 0.049). When analyzing the depressive symptomatology about gender and age, a significant association is identified with the increase in depression scores in the female gender (*p* < 0.001). Also, we can observe trends with higher values in the scores in the 30–59 age group. The complete values are observed in Table 4. Variables such as marital status, occupation, phototype or even the type of skin cancer were not associated with significant differences in personality or depression scores (*p* > 0.05).

**Table 4:** Personality dimensions, depression, and the influence of age and gender

		Gender			Age (years)			
	Female		Male		30–59		60–90	
Measure	Media ± SD	***Median***	Media ± SD	***Median***	Media ± SD	***Median***	Media ± SD	***Median***
*Depression total score*	39.8 ± 11.7 **‡	37.5	**29.3 ± 5.5﻿**	29.0	38.7 ± 12.6	**37.0**	34.18 ± 10.1	**33.0†**
*EPQ-R scores*								
*Extraversion*	**12.1 ± 4.4‡**	13.0	**11.1 ± 5.1﻿**	11.0	**11.2 ± 4.8**	10.0	**11.8 ± 4.7‡**	13.0
*Neuroticism*	14.8 ± 5.9	16.0*†	8.0 ± 5.3	**8.0﻿**	**11.9 ± 8.5**	11.5	**11.7 ± 6.1‡**	10.0
*Psychoticism*	**7.5 ± 2.4‡**	7.0	**6.2 ± 2.7﻿**	6.0	**6.3 ± 2.4**	6.5	**7.1 ± 2.7‡**	7.0
*Lie*	14.4 ± 3.1	**14.5†**	14.1 ± 2.5	**15.0﻿**	12.5 ± 3.4	**12.5**	14.7 ± 2.4	15.0*†

Notes: *SD* Standard deviation; † Mann–Whitney U test, ‡ Student’s t-test; *p < .05, **p < .01

Finally, the relationship between the scores of the dimensions of the EPQ-R questionnaire and depression scores was evaluated. The results show significant correlations between anxious and depressive symptomatologic portraits with most of the personality dimensions except E, obtaining positive correlations with N and P dimensions (*p* < 0.001, r = 0.705 and *p* = 0.003, r = 0.422), while a negative correlation was observed with dimension L (*p* = 0.028, r = − 0.321). Another interesting result were the correlations between the different personality dimensions E with P (*p* = 0.018, r = − 343), N with P (*p* < 0.01, r = 0.468) and P with L (*p* = 0.026, r = − 0.325). The results are detailed in Table 5.

**Table 5:** Correlations for measures of personality and depression in patients

Measure	1 CQDD	2 EPQ-E	3 EPQ-N	4 EPQ-P	5 EPQ-L
*1.Questionnaire for depression (CQDD)*	(0.86) a				
*2. EPQ-Extraversion (EPQ-E)*	−0.172*‡*	(0.84) a			
*3. EPQ-Neuroticism (EPQ-N)*	0.705 **†	−0.118*†*	(0.91) a		
*4. EPQ- Psychoticism (EPQ-P)*	0.422**‡	−0.343*‡	0.468**†	(0.49) a	
*5. EPQ-Lie (EPQ-L)*	−0.321*‡	0.111*‡*	−0.246 *†*	−0.325*‡	(0.68) a

Notes: † Pearson’s r or ‡ Spearman’s correlation coefficients; a Cronbach’s alpha; *p < .05, **p < .01. Numbers 1–5 correspond to the five measures listed

## Discussion

Skin cancer is characterized by being a disease in which there is an abnormal growth of the cells of the skin tissues. Represents the most common cancer worldwide, exceeding the prevalence of other types of malignant neoplasms combined. It is classified in melanoma and non-melanoma skin cancer (NMSC), the latter encompassing keratinocyte carcinomas which can be subclassified into BCC and SCC. NMSC represents 99% of the total of skin neoplasms [24–26]. Most of the patients included in our study present BCC (70.2%), followed by SCC and with very few patients with mixed carcinoma and melanoma. This coincides with various registries that show that the prevalence is higher in BCC than in any other type of skin cancer [27, 28]. In addition, a higher percentage of skin cancer has been reported in men compared to women, with values ranging from 60 to 63% [29, 30]. In the study, a similar amount was presented in both genders; the differences may be influenced by the type of sampling and the number of patients included.

It has been previously reported that the incidence of NMSC increases with age, the probability of presenting BCC / SCC with each year of age increases up to 10%, while in melanoma only 4%, this could be since the older the immune system is less efficient in the resolution of some DNA damage [31]. Based on the results of the present study, it was identified that the mean age of patients with skin cancer is 66.5 years and that the age group with the highest percentage is 60–90 years. These results are like various studies carried out in a European population, with means of 63 years of age in patients with BCC and 64.7 years for patients with SCC [28, 29]. While the age range with the highest percentages has been described in patients with skin cancer is 61 to 70 years [32, 33].

Although, there are previous studies that refer to an occupation as a risk factor, finding a higher prevalence in the work of welders, specialized farmers, miners, and stonemasons in patients with BCC. While in those with SCC, occupations are to a greater extent than involved direct contact with livestock, construction workers, stationary motor operators, and bricklayers [28, 30]. Meanwhile, in our study, the highest percentage of patients reported being housewives. Other factors that could be influencing are the FST and the degree of solar exposure; The latter being one of the factors with the greatest impact on human skin and is particularly important in terms of sensitivity to UV-induced DNA damage [34, 35]. As in previous studies, the results of this study identified most of the participants with FST II and III. FST is one of the factors identified with the development of skin cancer, finding a higher prevalence in populations with light skin phototypes. In addition, it has been widely described that the location of skin cancer lesions is to a greater extent in photo-exposed areas such as the head, neck, cheeks, nose, etc. [32, 33]. These data agree with those found in the study population included, finding that 68.2% of skin cancers are in the head or neck region.

Regarding the results obtained for the personality dimensions. Taking into consideration the measures provided by the EPQ-R manual applied in healthy population for the different dimensions with means of 12.7 ± 4.0 for E, 13.4 ± 5.4 for N, 5.7 ± 3.5 for P, and 8.4 ± 3.8 for L. We observe similar values except for L, which is above the mean. However, this differential behavior may be due to the age of the subjects included, as is previously reported that L scores are higher in ages between 40 to 60 years. However, it is not the only dimension that has been associated with age, E is also associated even though it is inversely associated [36, 37].

The link between personality and cancer development has not yet been demonstrated, with conflicting results in different types of tumors. According to the results of some investigations where personality was evaluated in patients with lung, colon, breast, prostate, skin, leukemia, and lymphoma cancers, no associations were obtained regarding the risk of their development [20, 38]. However, there are reports of male patients with lung cancer presenting higher extraversion scores and lower neuroticism scores [39]. Others, for their part, have found in cancer survivors patients present higher levels of psychoticism and neuroticism with low levels of extraversion, although without significant differences [40]. Although the absence of a control group eliminates the possibility of inquiring about the possible connection between personality and the diagnosis of skin cancer in the subjects included. As in the studies mentioned above, our results reveal the influence of gender by obtaining higher values in the dimension of N in the female gender. The foregoing has been previously described in open populations, showing higher levels of N in women compared to men [41].

Environmental psychological factors such as mood states can modify and trigger an organic response [42, 43]. Personality relates to mood; some authors identify high scores in neuroticism associated with intense and long-lasting emotions of stress and therefore with the development of depressive behaviors [44]. It has been observed that cancer patients present comorbidities with psychological illnesses such as depressive disorder, even being considered as a risk factor for oncological development, leading to alterations both in the genesis and in the progression of the disease [3, 10, 45]. In addition, it has been documented that in older adults the presence of this disorder is associated with increased neuroticism in cancer survivors [46–48].

Measures of anxiety and depression and fear of cancer and negative associations with quality of life and emotional well-being in patients with cutaneous melanoma, especially in the female gender [16, 21, 49]. Similarly, in patients with BCC, a positive association with the presence of depression has been recorded [17]. Although NMSC places a greater burden of symptoms on patients than melanoma, the psychological impact of melanoma is higher [50]. Our results coincide with previous studies by identifying that more than a third of patients with skin cancer show anxiety/depressive symptoms. Also, we found that there is a significant relationship between depressive symptomatology scores and female gender. These results are highly relevant when knowing that depression is associated with just over half of the incidence of suicides, in addition to the fact that there are studies that mention a high prevalence of suicides in female patients with NMSC, it is commented that the deforming capacity of the disease as the main reason [27, 51]. Therefore, the detection and treatment of the disease in early stages acquires greater relevance in terms of skin cancer resolution and the psychological impact. Overall, patients who underwent dermatologic surgery on the face reported a low level of psychosocial distress [52].

Subsequently, we encounter that there are positive correlations between the scores for the anxious/depressive symptoms and the personality dimensions N and P. Something that has also been mentioned in previous studies where high levels of neuroticism can generate vulnerability to depressive symptoms. In addition, psychoticism has been shown as a predictor of depressive symptoms in breast cancer survivors [53, 54]. Furthermore, we observed positive correlations between the dimensions of N and P which have not been found in the reviewed literature. However, it has been identified that high levels of both N and P are related to negative coping responses to stress and suicidal ideation [37, 55, 56]. A negative correlation between anxiety/depression and the L dimension scores was also found, people with high L scores are related to the presence of psychiatric disorders such as depression and schizophrenia [36, 57].

Limitations of this study include its cross-sectional nature and the relatively small number of subjects studied. Therefore, we believe that further studies are required to confirm our results.

## Conclusions

Our study reinforced the high prevalence of BCC within the cases of skin cancer, the vulnerability according to age, and the influence of the phototype. On the other hand, the results obtained support the hypothesis that personality dimensions are related to the presence of anxiety/depressive symptomatology in patients with skin cancer, especially in the female gender. Highlighting the need for future research that delves into the implications at the psychological level, quality of life and the biological mechanisms that link personality and depressive symptoms in the development and evolution of skin cancer.

## Data Availability

Detailed data are available from the corresponding author upon request.
